# Religious and Spiritual Experiences, Discrimination, and Stress Among Midlife Women in the USA: The Study of Women’s Health Across the Nation

**DOI:** 10.1007/s10943-024-02189-z

**Published:** 2024-12-04

**Authors:** Marilyn J. D. Barnes, Imke Janssen, Sheila A. Dugan, Howard M. Kravitz, George Fitchett

**Affiliations:** 1 Department of Patient Counseling, Virginia Commonwealth University, 900 E. Leigh Street, Suite 7009, Box 980664, Richmond, VA 23298 USA; 2 Department of Family and Preventive Medicine, Rush University Medical Center, Chicago, IL USA; 3 Department of Physical Medicine and Rehabilitation, Rush University Medical Center, Chicago, IL USA; 4 Department of Psychiatry and Behavioral Sciences, Rush University Medical Center, Chicago, IL USA; 5 Department of Religion, Health, and Human Values, College of Health Science, Rush University Medical Center, Chicago, IL USA

**Keywords:** Perceived discrimination, Stress, Religion, Spiritual experiences

## Abstract

The harmful effects of perceived discrimination for physical and mental health are well documented. Evidence identifies how dimensions of religious/spiritual (R/S) involvement may reduce these harmful effects. This study examined how R/S experiences are associated with the effects of discrimination on perceived stress. With data from the Study of Women’s Health Across the Nation (SWAN), we examined the offsetting and buffering effects of daily spiritual experiences on the relationship between everyday discrimination and perceived stress among 2,221 US midlife women from 5 racial/ethnic groups. Regression analysis identified a positive association between perceived discrimination and perceived stress (*p* < .001). Daily spiritual experiences were inversely associated with perceived stress (*p* < .001) for the whole sample and in the subsample of Black women. For this subsample, there was an inverse association between daily spiritual experiences and discrimination. R/S experiences may be one of the ways that R/S reduce the harmful effects of discrimination on health.

## Introduction

Perceived discrimination is a widely recognized stressor, and its harmful effects on mental and physical health for women and men of various races are well documented (Barnes et al., 2008; Lewis et al., 2015; Pasco & Smart Richman, 2009; William & Mohammed, 2009). A large systematic review and meta-analysis of racism, including self-reported personal and vicarious experiences of racism, found associations with poorer general, mental, and physical health (Paradies et al., 2015). Among middle-aged women from 5 racial/ethnic groups who participated in the Study of Women’s Health Across the Nation (SWAN), perceived discrimination was associated with higher allostatic load (Upchurch et al., 2015) and greater reports of bodily pain (Dugan et al., 2017). Among these women, perceived discrimination was also associated with greater incidence of metabolic syndrome and increased BP over time (Moody, et al., 2019).

Compared to other racial/ethnic groups, there is evidence that Black men and women report higher levels of perceived discrimination (Barnes et al., 2004; Sylvers et al., 2022). Among the women in the SWAN study, Black women reported the highest levels of unfair treatment (65%) (Brown et al., 2006). Research also shows that among Black women and men, perceived discrimination is associated with poorer mental and physical health (Black et al., 2015; Lewis et al., 2015; Williams & Mohammed, 2009). This includes evidence of an association with coronary artery calcification among Black women who participated in the SWAN heart study (Lewis et al., 2006).

For more than 25 years, investigators have been examining the association between religion/spirituality (R/S) and health (Levin, 1996). Spirituality is defined as a dynamic and intrinsic aspect of humanity through which persons seek ultimate meaning, purpose, and transcendence, and experience relationship to self, family, others, community, society, nature, and the significant or sacred (Puchalski et al., 2014). Religion is the search for significance that occurs within the context of established institutions that are designed to facilitate spirituality (Hill et al., 2000). A substantial body of research documents the positive association between R/S involvement and health. A recent systematic review of 276 studies with low risk of bias found evidence that frequent attendance at public worship was associated with better mental and physical health outcomes, better quality of life, and better health behavior (Balboni et al., 2022).

Investigators have described several mechanisms that may account for these positive effects, including better health behaviors and greater social support among people with higher levels of R/S involvement (Park et at., 2017). It has also been suggested that R/S involvement may improve host resistance to the harmful effects of stress (Ellison & Fan, 2008; Levin, 1996). However, it should be noted that other studies have described the harmful effects of R/S struggles (e.g., religious doubts) on health, including evidence they may mediate the association between day-to-day discrimination and depression (Exline et al., 2014; Hill et al., 2017).

R/S are multi-dimensional and include beliefs, private and group devotional practices, and relationships (Idler et al., 2003; Park et al., 2017). R/S experiences are another important dimension of R/S and may include feeling close to God or other divine beings (Ellison et al., 2008; Underwood & Teresi, 2002). R/S experiences may also play an important role in buffering the harmful effects of stress; “Experiences of God’s presence and guidance may reduce feelings of psychological stress, thereby moderating the link between social stressors and health and wellbeing” (Ellison & Fan, 2008). Evidence for this relationship was found in a study of 2,967 Black women and men who participated in the Jackson Heart Study, in which higher scores on the Daily Spiritual Experiences scale were associated with lower levels of global perceived stress (Brewer et al., 2022). It is important to note there are two models for the relationship between health, stress and R/S experiences, or any dimension of R/S (Ellison & Fan, 2008). In the first model, the positive effects of R/S *offset* the negative effects of stress on health. Statistically, this model is usually tested by examining the independent main effect of R/S on health in models that also include the main effects of stress. In the second model, the negative effects of stress are assumed to vary with or are *buffered* by R/S, being less harmful for those with higher levels of R/S involvement. This model is usually tested with a R/S by stress interaction term in models controlling for the main effect of R/S and stress.

A substantial body of evidence describes high levels of R/S involvement for Black people. In a national Pew survey, Blacks report greater weekly worship attendance and daily prayer than Hispanics or Whites (Pew Research Center, 2021). Another large national study found higher levels of religious coping (e.g., look to God for strength, support, and guidance) among Blacks compared to non-Hispanic Whites (Chatters et al., 2008). This and other studies (Brewer et al., 2022) have also reported higher levels of R/S involvement and R/S coping among Black women compared to Black men. Among the women who participated in SWAN, Black and Hispanic women had similarly high levels for 5 measures of R/S involvement compared to the White, Chinese, and Japanese women (Fitchett et al., 2007).

In light of the harmful effects of discrimination on health for Blacks, investigators have examined whether R/S involvement may have a protective effect. This protective effect may come from different dimensions of R/S including religious social support and R/S beliefs about dignity and divine control (Upenieks & Daniels, 2022). Previous research reports that worship attendance and religious social support buffer the effects on depression of unfair treatment by police for Blacks but not Whites (Upenieks & Daniels, 2022). Among Blacks, religious social support buffered the effects of discrimination on depression and life satisfaction; that is increased experiences of discrimination were associated with greater depression for those with low but not for those with high levels of religious social support (Ellison et al., 2017). Another study among Black women and men found that frequent worship attendance and religious guidance buffered the harmful effects of discrimination on psychological distress while religious social support offset these effects (Ellison & Fan, 2008).

In the present study, we extend this research by focusing on the role of R/S experiences in reducing the harmful effects of perceived discrimination on health. Specifically, we examine two possible models of stress reduction, offsetting and buffering, for the effects of R/S experiences on the association between perceived discrimination and perceived stress. We examine these associations in a multiracial/ethnic sample of midlife women. Considering the harmful effects of racism on Blacks and their high levels of R/S involvement, we also examine these models of stress reduction for R/S experiences among Black women who report experiences of racism.

## Methods

### Participants

Data for this study came from the Study of Women’s Health Across the Nation (SWAN), a study of health and aging in midlife women across the menopausal transition. For this study, we used data from the baseline and follow-up years 1 through 4 (1997–2002). SWAN annual interviewer-administered surveys were conducted in 7 sites in the USA (Boston, Chicago, Southeast Michigan, Los Angeles, Newark, Oakland, CA, and Pittsburgh). The entire cohort consisted of 3,302 racially/ethnically diverse midlife women who at the start of the study ranged in age between 42 and 52 years old. Details of the study have been described elsewhere (Sowers, et al., 2000). In SWAN, each site recruited White women and a predetermined minority group (Black women in Boston, Chicago, Detroit, and Pittsburgh, Chinese women in Oakland, Hispanic women in Newark, and Japanese women in Los Angeles). Eligibility criteria also included having an intact uterus and at least one ovary and having had at least one menstrual period and no use of reproductive hormones and not pregnant or breastfeeding/lactating in the previous 3 months. Institutional review board approval was obtained at each SWAN study site, and written informed consent was obtained from participants.

For this study, baseline was defined as the first visit at which the Daily Spiritual Experience scale (DSE) (Underwood & Teresi, 2002) was administered, which was during follow-up year 4 of the SWAN study (January 2000 to March 2001). The sample consisted of 2,655 participants who responded to the DSE and the perceived stress questions in follow-up year 4. Participants missing measures of everyday discrimination or daily spiritual experience were excluded bringing the sample size to 2,221 participants for the multiracial/ethnic sample. Among Black women, 427 (68% of the Black women in the study) reported race/ethnicity as the main source of discrimination; this is the sample we used for that analysis.

### Measures

Perceived stress was the main outcome variable and was measured using 4 items from the perceived stress scale (PSS4). The 4-item version of the scale has been validated for studies that require shorter instruments (Cohen et al., 1983). Participants indicated the frequency with which they experienced four stressful situations using a 5-point Likert scale (1 = never, 5 = very often). For scoring total perceived stress, responses to the 2 negative questions were summed with the reverse of the responses to the 2 positive questions, yielding a composite score ranging from 4 to 20. Higher PSS4 scores indicated increased time experiencing stressful situations in the prior two weeks.

Perceived discrimination was measured with the 10-item everyday discrimination scale (EDS) (Williams et al., 1997). The EDS measures day-to-day experiences of unfair treatment and how often respondents felt discriminated against. The responses were reverse coded with 1 being equal to “never” and 4 being equal to “often” so that the higher scores correspond to higher levels of perceived discrimination. We calculated a measure of chronic discrimination (Lewis et al., 2013) by creating an average score across the baseline and year 1–3 visits (potential score is 1–4). The EDS also asks participants who responded “often” or “sometimes” to any of the 10 questions to provide the reason for their perception of the discrimination. In the version of the EDS used in follow-up visits 1–3, there are ten choices including race, ethnicity, gender, age, income level, language, body weight, physical appearance, sexual orientation, and other. In the version of the EDS used in the baseline survey, the question asked for the “main reason” for the experiences with the same choices.

R/S experiences were measured with an 8-item version of the Daily Spiritual Experience scale (DSE) (Underwood & Teresi, 2002). The DSE was created to measure the experience of connection to God or the transcendent and other everyday experiences and feelings that grow out of that experience of connection (Underwood & Teresi, 2022). The DSE has 16 items. SWAN included a version with 8 items: 2 items assessing closeness with God (“I feel God’s presence.” “I desire to be closer to, or in union with God.”), 2 items assessing interpersonal experiences (“I feel a selfless caring for others.” “I accept others even when they do things that I think are wrong.”), and 4 items assessing feelings (“I feel a deep inner peace or harmony.” “I find comfort in my religion/spirituality.” “I am spiritually touched by the beauty of creation.” “I am thankful for my blessings.”). In a sample of Caucasian and Black women from the Chicago site of SWAN, the correlation between this 8-item version of the DSES and the full 16-item version was 0.96. Participants with valid responses to 6 of the 8 items were included in the analysis. The average of the non-missing items was used for imputation. The possible responses range from 1 for “many times a day” to 6 for “never or almost never.” The responses were reverse coded so that higher scores correspond to greater frequency of R/S experiences. Potential scores could range from 8 to 48.

Study covariates included age, race, education, marital status, children, financial strain, and study site. Marital status was coded dichotomously, married (or living together as married) or not married. Children was coded dichotomously, yes or no. Race was coded in 5 categories: Black, White, Chinese, Hispanic, and Japanese. Education was coded in 3 categories: college degree or greater, some college, and high school or less. Financial strain was measures with the item “How hard is it to pay for basics?” Responses were dichotomized: very or somewhat hard vs not very hard. Study site was coded aligning with the seven study sites. To characterize the sample, both the whole multiracial/ethnic sample and the subsample of Black women, we included 2 other measures of R/S involvement: R/S salience and R/S coping.

### Analysis

The analysis began with the calculation of descriptive statistics for the study measures. We then tested the correlation among the main study measures, perceived discrimination, daily spiritual experiences, and perceived stress. We used a series of linear regression models to examine the relationship among perceived discrimination, daily spiritual experiences, and perceived stress. The first model, testing the *offsetting* effect of R/S experiences, included the main effects of perceived discrimination and daily spiritual experiences. In the second model, we added a perceived discrimination by daily spiritual experienced interaction term to test the *buffering* effects of R/S experiences. We repeated these models with adjustment for the covariates. These analyses were conducted separately for the whole multiracial/ethnic sample and the subsample of Black women who reported race/ethnicity as the reason for perceived discrimination. All analyses were conducted utilizing SAS software (Version 9.4).

## Results

The demographic characteristics of the study sample, both the multiracial/ethnic sample of all women and the subsample of Black women who reported experiencing racial discrimination, are shown in Table 1. As can be seen in the table, among the women in the multiracial/ethnic sample, the average age was 49.9 years. By design, approximately half the women reported White race (49.2%) and slightly more than one-fourth (28.4%) reported being Black. Almost two-thirds reported being married (63.3%) and more than three-fourths had children (83.5%). More than three-fourths of the women (77.3%) had at least come college education and just over three-fifths (61.6%) reported it was not hard to pay for basics. Among this sample, the mean perceived stress score was 7.9 (SD = 3.0), the mean everyday discrimination score was 1.7 (SD = 0.4), and the mean daily spiritual experience score was 33.7 (SD = 8.6).

**Table 1:** Sample characteristics: all women (*N* = 2221) and subsample of Black women (*N* = 427)*

	All women *N* (%)	Black women *N* (%)
*N*	2221	427
Age at Outcome, yrs, mean (SD)	49.9 (2.7)	49.8 (2.6)
*Race/Ethnicity*		
White	1093 (49.2)	
Black	631 (28.5)	
Japanese	186 (8.4)	
Chinese	145 (6.5)	
Hispanic	166 (7.5)	
Married, *N* (%)	1406 (63.3)	192 (45.0)
Any children, *N* (%)	1854 (83.5)	380 (89.0)
*Education, N (%)*		
College +	983 (44.3)	149 (34.9)
Some college	733 (33.0)	184 (43.1)
HS or less	484 (21.8)	87 (20.4)
Missing	21 (0.9)	7 (1.6)
*Financial strain, N (%)*		
No	1369 (61.6)	228 (53.4)
Yes	836 (37.6)	195 (45.7)
Missing	16 (0.7)	4 (0.9)
*Site*		
Ann Arbor, MI	351 (15.8)	139 (32.6)
Boston, MA	337 (15.2)	112 (26.2)
Chicago, IL	283 (12.7)	89 (20.8)
Alameda and Contra Costa County, CA	285 (12.8)	–
Los Angeles, CA	366 (16.5)	–
Jersey City, NJ	245 (11.0)	–
Pittsburgh, PA	354 (15.9)	87 (20.4)
Perceived stress, mean (SD) (potential score 4–20)	7.9 (3.0)	8.1 (3.2)
Everyday Discrimination Scale, mean (SD) (potential score 1–4)	1.7 (0.4)	2.1 (0.4)
Daily Spiritual Experiences scale, mean (SD) (potential score 8–48)	33.7 (8.6)	38.0 (6.9)

^*^Black women who report race as reason for discrimination

For the subsample of 427 Black women who reported racial discrimination (68% of all Black women in the study), the average age was 49.8 years (SD = 2.6). Less than half (45%) were married, and the vast majority (89%) reported having children. More than three-fourths (78%) had at least some college education and slightly more than half (53.4%) reported it was not hard to pay for basics. Among these women, the mean perceived stress score was 8.1 (SD = 3.2), the mean everyday discrimination score was 2.1 (SD = 0.4), and the mean daily spiritual experience score was 38.0 (SD = 6.9).

There were high levels of R/S for the total sample and the subsample of Black women. Among all the women, 61.5% reported that R/S is very important and 66.5% report R/S is a source of a great deal of strength and comfort. Among the Black women in the subsample, 83.1% report R/S is very important and 89% report R/S is a source of a great deal of strength and comfort.

As shown in Table 2, among the sample of all women and the subsample of Black women, perceived discrimination was significantly associated with higher levels of perceived stress (all women, *r* = 0.192; Black women, r = 0.259). In both groups, scores on the Daily Spiritual Experiences scale were inversely and significantly associated with perceived stress (all women, *r* = − 0.157; Black women, r = − 0.199). Among the Black women, but not the sample of all women, scores on the Daily Spiritual Experiences scale were also significantly and inversely associated with perceived discrimination (r = − 0.157).

**Table 2:** Pearson correlations between perceived stress, discrimination, and daily spiritual experiences for all women (*n* = 2,221) and the subsample of Black women (*N* = 427)

	All women		Black women	
	Perceived stress	Daily spiritual experience	Perceived stress	Daily spiritual experience
Everyday Discrimination Scale (EDS)	0.192***	− 0.025	0.259***	− 0.157**
Daily Spiritual Experience scale (DSE)	− 0.157***		− 0.199***	

^**^p < 0.01; ***p < 0.001

Tables 3 and 4 present the results of the regression analysis for perceived stress for both the total sample (Table 3) and the subsample of Black women (Table 4). In both samples, in unadjusted and adjusted models (Model 1 and Model 3) there are significant positive associations between perceived discrimination and perceived stress and significant inverse associations for perceived stress and the DSE scores. The tables also show that the interaction between perceived discrimination and DSE scores are non-statistically significantly associated with perceived stress in both unadjusted and adjusted models (Model 2 and Model 4).

**Table 3:** Linear regression analyses for perceived stress: all women (N = 2221)

	Model 1	Model 2	Model 3	Model 4
Variable	Estimate (95% CI)	Estimate (95% CI)	Estimate (95% CI)	Estimate (95% CI)
Intercept	**8.46 (7.69, 9.23)**	**7.33 (5.39, 9.28)**	**8.52 (6.21, 10.82)**	**7.80 (4.90, 10.71)**
EDS	**1.64 (1.35, 1.93)**	**2.30 (1.22, 3.38)**	**1.51 (1.22, 1.80)**	**1.92 (0.87, 2.97)**
DSE	** − 0.05 ( − 0.07, − 0.04)**	− 0.02 ( − 0.07, 0.03)	** − 0.06 ( − 0.07, − 0.04)**	− 0.04 ( − 0.09, 0.02)
EDS * DSE		− 0.02 ( − 0.05, 0.01)		− 0.01 ( − 0.04, 0.02)
Age			− 0.01 ( − 0.05, 0.03)	− 0.01 ( − 0.05, 0.03)
Married			** − 0.30 ( − 0.56, − 0.05)**	− 0.30 ( − 0.56, − 0.05)
Children			0.05 ( − 0.28, 0.37)	0.05 ( − 0.27, 0.38)
Financial strain				
Yes			**1.15 (0.89, 1.40)**	**1.14 (0.89, 1.40)**
Missing			**2.25 (0.69, 3.81)**	**2.24 (0.68, 3.79)**
Education				
Some college			0.12 ( − 0.15, 0.39)	0.12 ( − 0.15, 0.39)
< = HS			**0.58 (0.26, 0.91)**	**0.58 (0.25, 0.90)**
Missing			− 0.03 ( − 1.40, 1.35)	− 0.03 ( − 1.40, 1.35)

Models are adjusted for race/ethnicity and study site

Financial strain: reference group = no

Education: reference group = College +

EDS, Everyday DiscriminationSscale; DSE, daily spiritual experiences scale

Bold: significant at *p* < 0.05

**Table 4:** Linear regression analysis for perceived stress: subsample of Black women (N = 427)

	Model 1	Model 2	Model 3	Model 4
Variable	Estimate (95% CI)	Estimate (95% CI)	Estimate (95% CI)	Estimate (95% CI)
Intercept	**7.11 (4.75, 9.46)**	3.41 ( − 4.77, 11.6)	**8.57 (2.49, 14.66)**	5.50 ( − 4.9, 15.90)
EDS	**1.81 (1.10, 2.52)**	3.52 ( − 0.18, 7.21)	**1.74 (1.02, 2.46)**	3.06 ( − 0.64, 6.76)
DSE	** − 0.07 ( − 0.12, − 0.03)**	0.02 ( − 0.19, 0.24)	** − 0.08 ( − 0.12, − 0.04)**	0 ( − 0.21, 0.21)
EDS* DSE		− 0.05 ( − 0.14, 0.05)		− 0.04 ( − 0.13, 0.06)
Age			− 0.04 ( − 0.15, 0.07)	− 0.04 ( − 0.15, 0.07)
Married			− 0.03 ( − 0.62, 0.56)	− 0.03 ( − 0.62, 0.57)
Children			0.35 ( − 0.56, 1.27)	0.41 ( − 0.52, 1.33)
Financial strain				
Yes			1.17 (0.58, 1.76)	1.17 (0.58, 1.76)
Missing			4.60 (1.64, 7.56)	4.56 (1.60, 7.53)
Education				
Some college			0.01 ( − 0.65, 0.66)	0.01 ( − 0.64, 0.66)
< = HS			0.54 ( − 0.28, 1.36)	0.53 ( − 0.29, 1.35)
Missing			0.75 ( − 1.55, 3.04)	0.77 ( − 1.53, 3.07)

Models are adjusted for study site

Financial strain: reference group = no

Education: reference group = college +

Bold: significant at *p* < 0.05

## Discussion

Our findings provide evidence that in a multiracial/ethnic sample of midlife women R/S experiences offset the harmful effects of perceived discrimination on perceived stress. We further show that such R/S experiences offset the harmful effects of perceived discrimination on perceived stress for Black women who attribute their experiences of discrimination to racism. In both groups of women, there was no support for R/S experiences buffering of the effects of perceived discrimination; that is, we found no evidence that these effects varied by level of R/S experiences. We also found evidence, among the Black women only, that R/S experiences were inversely associated with perceived discrimination.

Compared with other measures of R/S involvement, research about R/S experiences and health is somewhat newer and less common (Balboni et al., 2022). However, the DSE Scale was included in the Jackson Heart Study (JHS), a study of risk for cardiovascular disease in over 5,000 Black men and women in Mississippi (Brewer et al., 2022). In the JHS, the DSE scores for Black women are very similar to those for the Black women in the present study and those scores were inversely associated with scores on the Global Perceived Stress Scale which includes discrimination (Brewer et al., 2022).

A moderate body of research has examined whether various dimensions of R/S reduce the harmful effects of discrimination on health. Most of these studies have examined these associations among Black women and men but one study used a sample of White and Black midlife women and men (Bierman, 2006). This study found that worship attendance buffered the effects of discrimination on negative affect but only for the Black participants. The present study extends this research by examining these associations in a multiracial/ethnic sample of midlife women.

Many of the studies that examine whether R/S buffer the effects of discrimination in Black women and men have focused on psychological outcomes such as depression. However, a few studies have found buffering effects of R/S for physical health outcomes such as heart rate variability (Cooper et al., 2014), C-reactive protein (Droloet & Lucas, 2020), and the cortisol/DHEA ratio (Lee et al., 2021). Future research should include other measures of health that may be proximate to discrimination-related distress including allostatic load.

Among the studies of psychological outcomes among Black women and men, the evidence differs about which dimensions of R/S have an effect and whether the effect is offsetting or buffering. For example, Upenieks and Daniels found that worship attendance and church-based social support, but not belief in divine control, buffered the effects of unfair treatment by police on depression (Upenieks & Daniels, 2022). In contrast, Ellison and colleagues found that R/S social support but not worship attendance or prayer buffered the effects of discrimination on depression and life satisfaction (Ellison et al., 2017), while Nguyen and colleagues found that church-based support did not buffer the effects of discrimination on psychological distress in a sample of Black men (Nguyen et al., 2018). To these findings, our study adds information about the role of R/S experiences in the discrimination-health association. Additional research is needed to clarify the findings in this literature about the role of different measures of R/S in offsetting or buffering the discrimination-health association.

A new finding in our study is that among Black women, but not the whole multiracial/ethnic sample, DSE scores were inversely associated with perceived discrimination. In the stress-coping framework (Major et al., 2002), a key factor is the primary appraisal of the level of threat associated with a stressor. One possible interpretation of this inverse association between R/S experiences and perceived discrimination is that the Black women in the study with higher levels of R/S experiences do not perceive discrimination being as threatening as women with lower levels of R/S experiences. This may be because higher levels of R/S experiences reflect other R/S beliefs such as greater forgiveness of others or focusing on divine love for oneself, beliefs which may contribute to minimizing perceived discrimination (Lewis et al., 2015). Additional research is needed to replicate these findings regarding R/S experiences in other samples and to clarify possible mechanisms for their effects.

The strengths of SWAN include a large sample that is diverse in terms of geography as well as race and ethnicity. The sample was well-characterized. The data are comprehensive for the study participants, and the main variables were measured using well-established, valid, and reliable measures.

However, the finding of the present study should be interpreted in light of several limitations. The findings come from a sample of middle-aged women, and we cannot assume that they apply to younger or older women. Further, other research suggests caution in applying these findings to men. Specifically, compared to women, men in general and Black men in particular (Brewer et al., 2022) have lower levels of R/S involvement. In addition, gender differences have been observed in the buffering role of R/S attendance on the association between discrimination and sleep problems; the harmful effects of discrimination were evident for women with low worship attendance but there was no buffering among men (Bierman et al., 2018). There is also evidence that the harmful effects of discrimination on mental and physical health are stronger for women than men (Borrell et al., 2006). Another limitation is that several items assessing comfort and thankfulness may bias this version of the DSE, potentially increasing positive correlations with mental health outcomes (Koenig & Carey, 2024). In addition, the cross-sectional design of the study limits making any causal interpretations. While one longitudinal study suggests that discrimination precedes depression (Brown et al., 2000), further longitudinal research is needed to examine these relationships.

## Conclusion

There is strong evidence that discrimination has harmful effects on the health of women in general, including Black women. There is also emerging evidence that experiences of discrimination contribute to racial health disparities (Lewis et al., 2015). Like other dimensions of R/S, R/S experiences may offset those effects. Among Black women, these R/S experiences may contribute to reframing or minimizing experiences of discrimination which may limit their harmful effects. Further research is needed to extend our understanding of the role which diverse aspects of R/S, including R/S experiences, beliefs, and social support, play in reducing the harmful effects of discrimination on physical and mental health.
